# A Broken Heart After Witnessing a Dying Heart: A Case of Takotsubo Cardiomyopathy

**DOI:** 10.7759/cureus.28752

**Published:** 2022-09-03

**Authors:** Magnus To, Yolanda Zhang, Alexander Tompkins, Ruby Chen, Kaustubh Patankar, Sandeep Sangodkar

**Affiliations:** 1 Internal Medicine, Riverside Community Hospital, Riverside, USA; 2 Family Medicine, ProMedica Monroe Regional Hospital, Monroe, USA; 3 Cardiology, Riverside Community Hospital, Riverside, USA

**Keywords:** reversible cardiomyopathy, stress-induced cardiomyopathy, broken-heart syndrome, stress-related cardiomyopathy, takotsubo cardioyopathy

## Abstract

Takotsubo cardiomyopathy is a form of non-ischemic cardiomyopathy characterized by transient systolic dysfunction. The prevalence of Takotsubo cardiomyopathy has been estimated to be about 2% overall but about 10% amongst women presenting with clinical manifestations of acute coronary syndrome. The overall mechanism of the disease still remains unclear. However, treatment of Takotsubo cardiomyopathy appears to be similar to congestive heart failure (CHF) medical management. This case highlights the classic presentation exhibited very similar to acute coronary syndrome and diagnostic criteria for Takotsubo (stress-induced) cardiomyopathy.

## Introduction

Takotsubo cardiomyopathy, also known as Broken-Heart Syndrome, started being reported in the 1990s by Japanese authors [1,2]. They found that Takotsubo cardiomyopathy patients were usually postmenopausal women who often developed signs and symptoms of acute coronary syndrome around the period of a strong emotional stressor [1,2]. These patients are often found to have no obstructive coronary artery disease but transient apical and midventricular wall motion abnormality on coronary angiography and ventriculogram [1-3]. More healthcare providers are becoming aware of the disease and more research is looking into elucidating the pathophysiology and mechanism of action of the disease. Although the disease is not completely understood at the moment, its treatment has appeared to settle on conservative management and focus on emotional or physical stress relief [4,5]. Takotsubo cardiomyopathy has been recognized by the American College of Cardiology and the American Heart Association as a unique form of reversible cardiomyopathy.

## Case presentation

A 60-year-old woman with a past medical history of anxiety and stomach ulcers presented to the hospital for an acute onset of 10/10 chest pain radiating to her back. She had witnessed her husband fall flat on his face. She immediately called for emergency medical services (EMS) who advised her to initiate cardiopulmonary resuscitation (CPR) because he likely had a cardiac arrest. However, due to her husband’s morbid obesity, she was unable to flip her husband over in order to start CPR. As EMS arrived and attempted to revive her husband, the patient started to have severe 10/10 chest pain in addition to being tearful with worsening anxiety as a result of experiencing the ordeal. Despite EMS’s efforts, they were unable to revive her husband and he was pronounced dead at the scene. Due to the patient’s new onset of chest pain, EMS decided to treat her with aspirin and nitroglycerin and transported her to the hospital. 

**Figure 1:**
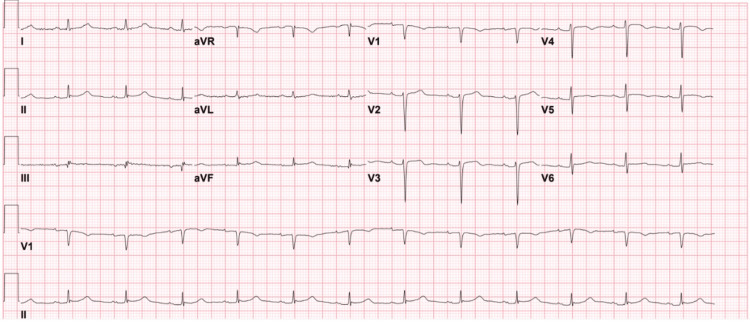
The patient’s ECG ECG shows normal sinus rhythm and normal axis with inverted T-waves in leads V4-V5 and flattened T-waves in leads III and V6.

The patient’s initial electrocardiogram (ECG) at the hospital showed normal sinus rhythm, heart rate 75/min, normal axis, QTc of 428ms, inverted T-waves in leads V4-V5 and flattened T-waves in leads III and V6 (Figure 1). 

High sensitivity troponins had an initial result of 584 that trended up to 4192 before down trending to 1650. She was hemodynamically stable while in the emergency room. Her chest X-ray during the initial evaluation in the emergency department was unremarkable. Computed tomography angiography (CTA) chest had ruled out a pulmonary embolism and aortic dissection. Her chest pain improved without receiving additional medications while in the emergency room and resolved when she was moved up to the medical/surgical floor. 

On collecting additional history from the patient, she endorsed an extensive family cardiac history including her mother who had a myocardial infarction at the age of 48 years followed by a quadruple coronary artery bypass graft at the age of 50 years, and her father who had multiple episodes of myocardial infarction and coronary artery disease also treated with a quadruple coronary artery bypass graft. Her paternal grandfather passed away from a myocardial infarction. Her paternal aunt and uncle had a history of myocardial infarction. Her maternal grandfather had a stroke.

**Figure 2:**
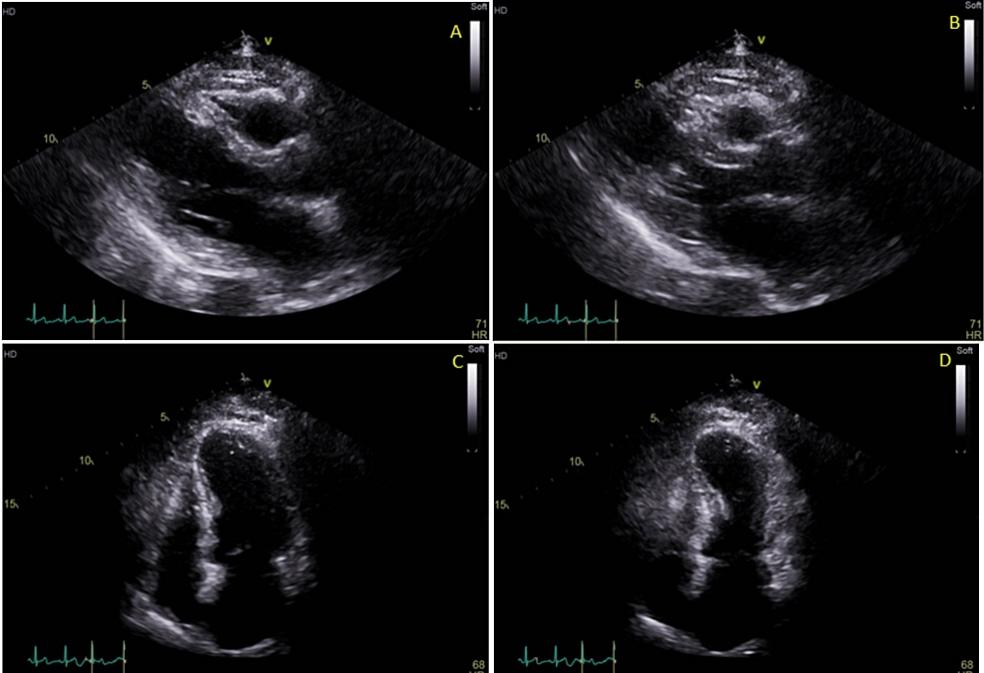
The patient’s transthoracic echocardiogram Echocardiogram images show apical akinesis and contraction of the mid and proximal portion of the left ventricle; (A) parasternal long axis at end-diastole (B) parasternal long axis at end-systole (C) apical four-chamber at end-diastole (D) apical four-chamber at end-systole.

A transthoracic echocardiogram (TTE) showed a left ventricular ejection fraction of 40-45% with basal hyperkinesis and apical akinesis suggesting stress-induced cardiomyopathy (Figure 2). 

**Figure 3:**
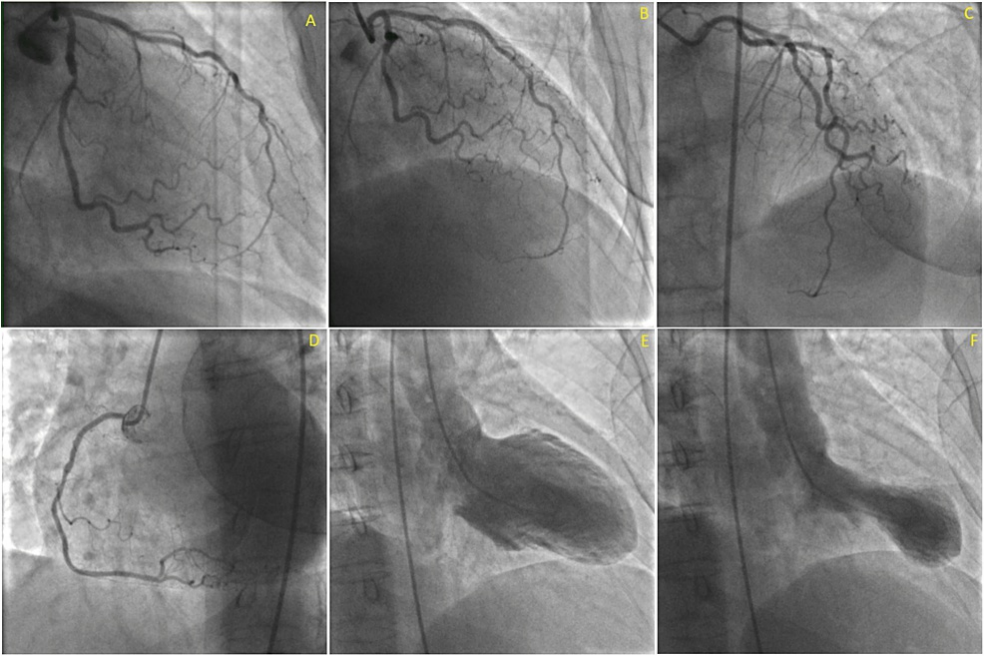
The patient’s cardiac catheterization images The patient’s cardiac catheterization images show lesions in the left circumflex artery – about 50-60% – in the RAO view (A), in the LAD – about 30% – in the RAO view (B), and in the right coronary artery – about 40% (D). RAO cranial view showing the LAD and left circumflex artery (C). The ventriculogram showed appropriate filling at end-diastole (E) and characteristic Takotsubo cardiomyopathy apical akinesia at end-systole (F). RAO: Right Anterior Oblique view; LAD: Left Anterior Descending Artery

Due to the increased risk factors, lab results, ECG findings, and TTE findings, cardiac catheterization was discussed with the patient who agreed to have the procedure done. Cardiac catheterization showed notable apical hypokinesis with normal left main coronary artery; left anterior descending had 30% stenotic lesion; left circumflex had 50-60% stenotic lesion; right coronary artery had 40% stenotic lesion (Figure 3). The lack of significant stenosis and apical hypokinesis were consistent with Takotsubo (stress-induced) cardiomyopathy. 

The patient was initiated on guideline-directed medical therapy including aspirin, atorvastatin, lisinopril, and metoprolol tartrate. Her chest pain had resolved and she was hemodynamically stable throughout hospitalization. She was discharged with recommendations to see a psychiatrist for her late husband and a cardiology follow-up for surveillance and monitoring. 

## Discussion

The term “takotsubo” comes from the name of a pot used to trap octopuses by Japanese fishermen. In Takotsubo cardiomyopathy, the neck of the ventricle contracts while the apex has minimal to no movements resulting in a shape similar to the octopus trap [6]. In a systemic review, women made up about 82-100% of Takotsubo cardiomyopathy cases with an average age ranging between 62-75 years but have been described in patients as young as age 10 and as old as age 91 [7]. Although some studies have estimated the prevalence to be 1-2%, the true prevalence and incidence remain unknown [1,3]. Since being reported in the 1990s in Japan, more cases of Takotsubo cardiomyopathy have been reported. Despite this, there is still some lack of awareness about the disease in even North America possibly lending to it being underdiagnosed [1].

The mechanism of Takotsubo cardiomyopathy, also colloquially known as Broken-Heart Syndrome, is thought to be induced by stress, occurring during periods of increased sympathetic drive resulting in increased catecholamine stimulation of the myocardium [1,2]. This phenomenon is also seen in cases of intracranial hemorrhages, strokes, and head trauma as well as other conditions like pheochromocytoma and critically ill patients [2]. The pathophysiology for the development of Takotsubo cardiomyopathy remains elusive but the hypothesized mechanism is the 2-3 fold elevation of plasma catecholamines and neuropeptides (norepinephrine, epinephrine, and dopamine) due to elevated levels found in patients with Takotsubo cardiomyopathy [7,8]. Increased catecholamine levels result in negative inotropy and left ventricular contractile dysfunction [7,8]. The apical aspect of the heart is hypothesized to have higher numbers of beta-adrenergic receptors [7]. This leads in more significant dysfunction at the apical aspect resulting in the characteristic apical akinesia. 

The diagnostic criteria of Takotsubo cardiomyopathy were proposed in 2004 to include: 1) transient akinesia or dyskinesia of left ventricular apex and mid-ventricular segments, 2) absence of angiographic evidence of acute plaque rupture or obstructed coronary arteries, 3) ECG abnormalities of either ST-segment elevation and/or T-wave inversion, and 4) absence of cranial trauma, intracranial bleeding, pheochromocytoma, myocarditis, and hypertrophic cardiomyopathy [9]. 

The disease frequently presents with chest pain and dyspnea resembling acute myocardial infarction but some patients may present with symptoms of acute decompensated heart failure like dyspnea on exertion, fatigue, weakness, and gravity-dependent pitting/non-pitting edema [1]. ECG may or may not present with ST-elevation, T-wave inversion, or Q-wave formation [1,5]. Patients most often present with elevated troponins followed by cardiac catheterization showing no flow-limiting lesions, which is often needed before a diagnosis of Takotsubo cardiomyopathy can be made. Cardiac MRI can be done to exclude other differential diagnoses like myocarditis or define any ventricular abnormalities that were poorly visualized on a transthoracic echocardiogram. 

Many suggest beta blockers for the blockade of the elevated catecholamines on the myocardium in Takotsuto cardiomyopathy. However, beta blockers do not always provide protection against recurrent Takotsubo cardiomyopathy [2,10]. Angiotensin-converting-enzyme inhibitors and angiotensin-receptor blockers have been associated with one-year improved survival [11]. Catecholamines should be avoided due to the sympathetic drive being the possible cause of the disease. Prognosis is generally favorable and most patients recover within weeks to months although few have experienced permanently reduced ejection fraction [2]. Some patients may experience complications due to the reduced ejection fraction. Mortality ranges between 1-2% for in-hospital patients with estimated recurrence rate to be 2-10% [2,12,13].

Our patient met all the criteria for the diagnosis of Takotsubo cardiomyopathy because she had ECG alteration (T-wave inversion), troponin level elevation, apical akinesia on transthoracic echocardiogram and no flow-limiting lesion on cardiac catheterization, with no known disease associated with elevated catecholamine or documented cranial pathologies. Although symptoms had resolved for our patient and she did not develop symptoms of acute decompensated heart failure, precautions and risk discussions were provided along with guideline-directed medical therapy and follow-up arrangements for psychiatric therapy for her late husband and for repeating the transthoracic echocardiogram in three months.

## Conclusions

Takotsubo cardiomyopathy or stress-induced cardiomyopathy results from left ventricular dysfunction that can present similarly to acute coronary syndrome or heart failure. Diagnostic criteria have been developed to better diagnose this form of cardiomyopathy. The prognosis is favorable with most patients recovering within weeks to months and few experiencing permanent dysfunctions. There is now better awareness from a physician's standpoint of Takotsubo cardiomyopathy. Patient education about the disease will create better awareness in order to improve avoidance of stressors that can result in recurrence or more harm.
